# Artificial intelligence in mammographic phenotyping of breast cancer risk: a narrative review

**DOI:** 10.1186/s13058-022-01509-z

**Published:** 2022-02-20

**Authors:** Aimilia Gastounioti, Shyam Desai, Vinayak S. Ahluwalia, Emily F. Conant, Despina Kontos

**Affiliations:** 1 Department of Radiology, Center for Biomedical Image Computing and Analytics (CBICA), University of Pennsylvania, Philadelphia, PA 19104 USA; 2 Mallinckrodt Institute of Radiology, Washington University School of Medicine, St. Louis, MO 63110 USA; 3 Perelman School of Medicine, University of Pennsylvania, Philadelphia, PA 19104 USA; 4 Department of Radiology, Hospital of the University of Pennsylvania, University of Pennsylvania, Philadelphia, PA 19104 USA

**Keywords:** Artificial intelligence, Deep learning, Breast cancer, Breast cancer risk, Breast density, Mammographic density, Digital mammography, Breast tomosynthesis, Mammographic imaging

## Abstract

**Background:**

Improved breast cancer risk assessment models are needed to enable personalized screening strategies that achieve better harm-to-benefit ratio based on earlier detection and better breast cancer outcomes than existing screening guidelines. Computational mammographic phenotypes have demonstrated a promising role in breast cancer risk prediction. With the recent exponential growth of computational efficiency, the artificial intelligence (AI) revolution, driven by the introduction of deep learning, has expanded the utility of imaging in predictive models. Consequently, AI-based imaging-derived data has led to some of the most promising tools for precision breast cancer screening.

**Main body:**

This review aims to synthesize the current state-of-the-art applications of AI in mammographic phenotyping of breast cancer risk. We discuss the fundamentals of AI and explore the computing advancements that have made AI-based image analysis essential in refining breast cancer risk assessment. Specifically, we discuss the use of data derived from digital mammography as well as digital breast tomosynthesis. Different aspects of breast cancer risk assessment are targeted including (a) robust and reproducible evaluations of breast density, a well-established breast cancer risk factor, (b) assessment of a woman’s inherent breast cancer risk, and (c) identification of women who are likely to be diagnosed with breast cancers after a negative or routine screen due to masking or the rapid and aggressive growth of a tumor. Lastly, we discuss AI challenges unique to the computational analysis of mammographic imaging as well as future directions for this promising research field.

**Conclusions:**

We provide a useful reference for AI researchers investigating image-based breast cancer risk assessment while indicating key priorities and challenges that, if properly addressed, could accelerate the implementation of AI-assisted risk stratification to future refine and individualize breast cancer screening strategies.

## Introduction

Randomized trials and screening cohort studies have clearly demonstrated that routine, mammographic screening is associated with a reduction in breast cancer morbidity and mortality [1]. Initially, breast cancer screening was performed with analog screen-film-based mammography systems, but over the last 20 years, mammographic screening has transitioned to fully digital platforms (full-field digital mammography (FFDM)) which allowed pixilated data to be reconstructed into the quasi-3D format of digital breast tomosynthesis (DBT) [2]. Additional efforts to improve breast cancer screening outcomes have focused on intensifying screening intervals and reading formats, e.g. yearly versus bi-annual screening and double-reading instead of single-reading, and introducing supplemental forms of screening in addition to mammography such as breast ultrasound or MRI [3]. In general, these enhanced screening protocols require additional resources and while they may detect more cancers, the additional imaging and increased intensity of screening may also result in higher false-positive rates [3]. As a result, there has been increasing advocacy for “personalized” breast cancer screening regimens, tailored to an individual women’s breast cancer risk based on a combination of imaging, demographic, and when available, genetic information [4]. Improvements in breast cancer risk assessment algorithms with the incorporation of image-derived data have the potential to help balance the harm-to-benefit ratios while better informing screening algorithms.

This complex landscape of mammographic screening offers several opportunities for improvements including the incorporation of computational imaging phenotyping of breast tissue. Importantly, doing so comes at little additional cost in terms of patient engagement and imaging time. For instance, mammographically assessed breast density, which reflects the amount of radio-dense tissue within the breast, has been well established as a risk factor for breast cancer as well as a feature that can reduce the sensitivity of mammography, since dense tissue may obscure or, “mask” tumors [5]. The recognition of breast density as a key biomarker in risk assessment has created a need for computational imaging efforts that deliver accurate and reliable measures of breast density areas, volumes and texture [6]. Recently, an array of computerized tools has been developed to convert mammographic images into phenotypic features for computational artificial intelligence (AI), commonly grouped under the umbrella of radiomic AI. The incorporation of breast radiomic features into breast cancer risk assessment algorithms has shown immense potential in improving breast cancer risk assessment and potentially, patient outcomes [7].

In the last 6 years, the computational medical imaging community has taken notice of an AI revolution driven by the introduction of deep learning (DL)-based convolutional neural networks (CNNs), which, compared to radiomic AI, possesses the advantage of ingesting images directly without explicit feature conversion [8]. These DL-based CNNs not only expanded the utility of imaging in predictive models but also pervaded breast cancer screening as one of the most promising computerized breast imaging tools. As in the title of this review, it is common to refer to AI, DL and CNNs almost interchangeably. However, AI generally refers to the creation of systems that perform tasks that usually require human intelligence, branching off into different techniques [9]. DL is one technique belonging to AI, and CNNs are only a subset of DL [9].

This narrative review synthesizes the current state-of-the-art applications of AI in mammographic phenotyping of breast cancer risk. For a more complete view of AI updates in breast cancer screening, we refer the reader to many excellent recent review papers on AI-enabled breast cancer detection [10] and broader applications of AI to breast imaging [11–13]. This review focuses on AI developments with the greatest potential to impact breast cancer risk assessment, specifically in the evaluation of digital 2D mammograms and 3D tomosynthesis images. We first briefly introduce key underlying concepts of AI and explore the advancements that led to the DL-driven revolution in computational medical imaging. Next, we focus on AI applications for assessing breast cancer risk from mammographic images, including breast density measurements as well as direct evaluation of breast cancer risk. Last, we discuss AI challenges that are unique to mammographic images and future directions for this promising research field.

## Main body

### The “wind of change” for AI in medical image computing

**Fig. 1:**
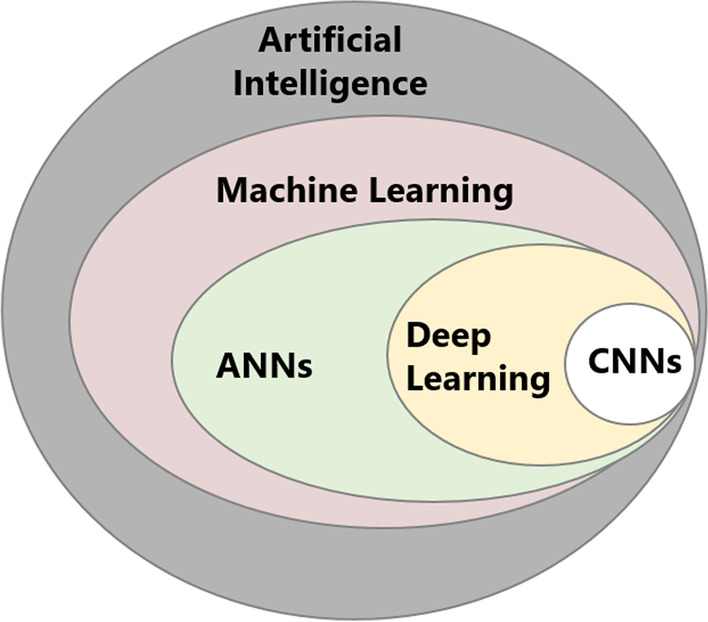
Diagram explaining the relationship between the different techniques in the field of artificial intelligence

AI is an umbrella term that encompasses various approaches to making machines mimic human decision-making (Fig. 1). Machine learning (ML) falls under the larger category of AI and includes all approaches that enable computers to learn from features extracted from training examples without those features being explicitly programmed [9]. Examples of ML approaches include regression, support vector machines, random forest classifiers, k-nearest neighbor algorithms, and artificial neural networks (ANNs) [9]. ML methods are divided into two broad paradigms: unsupervised learning and supervised learning [9]. Unsupervised learning aims to discover novel patterns in data that has no labels or categories assigned to training examples. The most common unsupervised learning task is clustering, which consists of grouping similar examples together according to predefined similarity metrics. In contrast, supervised ML methods train algorithms to classify data or predict outcomes by leveraging pre-labeled datasets. However, ML methods only work well if the input data contains meaningful predictive features from the start. Within ML, lies DL, which was developed to improve the performance of conventional ANNs using deep, multi-layered architectures [14]. Among the different deep ANNs, CNNs are based on convolutional operations that decode raw image data into complex data representations without needing to be explicitly fed image-derived features [14].

In general, the development of DL models requires a large amount of data for training, validation, and testing, with various imaging studies reporting logarithmic trends between model performance and data sample size [15]. However, the exact amount of data needed to achieve sufficient accuracy varies depending on the quality and variability of the data, as well as the DL model design, learning task and training approach [15–17]. The training set is usually the largest data set and is used to parameterize the model. The validation data set consists of data withheld from training that is used to further optimize the model’s hyper-parameters. Finally, the independent testing data set is used to determine performance benchmarks. DL and CNNs are not new concepts. Historically, training deep CNNs was considered impractical due to the limited availability of necessary data coupled with high computational costs. These challenges have been alleviated today because improved computational resources (such as advanced graphics processing units) and large data sets are becoming increasingly available. These computational advancements, along with the development of pivotal DL algorithms and training methodologies [18, 19], have brought DL to the mainstream in medical image computing, including applications assessing mammographic imaging data for breast cancer risk assessment.

### AI studies demonstrating robust and reproducible breast density assessment for improved risk estimation

The most commonly used method to assess breast density in the clinical setting is the visual and subjective grading of breast density by the interpreting radiologist into one of 4 categories outlined by the American College of Radiology (ACR) Breast Imaging-Reporting and Data System (BI-RADS) [20]. However, it has been well-established that a large degree of inter- and intra-reader variability exists in the assignment of breast density, particularly among less-experienced readers, with κ statistics ranging from 0.4 to 0.7 [21]. Furthermore, density categories were initially based on approximating the percent area of dense tissue in relation to the whole breast area (BI-RADS fourth edition, 2003 [22]), however, recently (BI-RADS fifth edition, 2013 [20]), the categories are no longer defined by percent density but rather the potential for masking of cancers by dense breast tissue. This change in BI-RADS definitions for the visual assessment of breast density has led to an increased number of women assigned to heterogeneously or extremely dense breast categories [23].

Despite the large inter- and intra-reader variation in BI-RADS density assessments, using them as the gold-standard in AI density models is a common approach, mainly due to the lack of large datasets with ground-truth density estimations. Actual ground-truth density estimations could be obtained only via breast excisions, while manual density segmentations are extremely time-consuming. Therefore, BI-RADS density assessments are usually the only ground-truth density information available for large mammographic datasets. Of note however is that despite the variability in subjective BI-RADS density assignments, they still remain highly predictive of future breast cancer risk [24].

To enhance reproducibility in breast density assessment, several studies have developed DL models of various architectures that learn to automatically classify mammographic images into BI-RADS density categories, using radiologists’ assessments [25–35] (Table 1). For instance, using raw (i.e., ‘For processing’) FFDM images from 1427 women, Mohamed et al. [33] applied transfer learning to develop a DL approach based on the AlexNet architecture. Their model achieved an AUC of 0.94 in BI-RADS density classification. Subsequently, using a separate dataset of 963 women, the authors demonstrated that the model performance varies by FFDM view type, with higher accuracy in mediolateral oblique (MLO) views (AUC = 0.95) than in craniocaudal (CC) views (AUC = 0.88) [34]. Then, using a substantially larger cohort consisting of processed (i.e., ‘For presentation’) FFDM images from 39,272 women, Lehman et al. [32] developed another DL model based on the ResNet-18 architecture and reported good agreement with 12 radiologists (four-class kappa (*K*) = 0.67). In the same paper, the DL model was evaluated in a reader study with five radiologists working in consensus on 500 FFDM exams randomly selected from the test set (four-class *K* = 0.78), and was also implemented in routine clinical practice, where eight radiologists reviewed 10,763 consecutive FFDM exams assessed with the DL model (four-class *K* = 0.85). Recently, the authors implemented their DL model at a partner community breast imaging practice and reported a high clinical acceptance rate among both academic (94.9%) and community (90.7%) radiologists as well as a reduction in the proportion of mammograms assessed as dense from 47 to 41% (*P* < 0.001) [25].

**Table 1:** Representative studies in AI-enabled breast density evaluation from mammographic images

Study	Model development dataset			Model design			Model performance
	Image format	# images (# women)	Vendors (# sites)	Model architecture	Output density measure	Density maps	
Roth et al. [35]	FFDM (Processed)	109,849 images (N/R)	N/R (7 sites)	DenseNet-121	BI-RADS density	No	Four-class *K* = 0.62–0.77
Dontchos et al. [25]	FFDM (Processed)	N/R (2174 women)	Hologic (1 site)	ResNet-18	BI-RADS density (13 interpreting radiologists)	No	Dense versus non-dense Acc: 94.9% (academic radiologists) 90.7% (community radiologists)
Matthews et al. [26]	FFDM (Processed) and SM	*FFDM*: 750,752 images (57,492 women) *SM*: 78,445 images (11,399 women)	Hologic (2 sites)	ResNet-34	BI-RADS density (11 interpreting radiologists)	No	Four-class *K* = 0.72 for FFDM, Site 1 Four-class *K* = 0.72 for SM, Site 1 Four-class *K* = 0.79 for SM, Site 2
Saffari et al. [27]	FFDM	410 images (115 women)	Siemens (1 site)	cGAN, CNN	BI-RADS density	Yes	DSC = 98% in dense tissue segmentation
Deng et al. [28]	FFDM	18,157 images (women)	Hologic (1 site)	SE-Attention CNN	BI-RADS density	No	Acc = 92.17%
Perez Benito et al. [29]	FFDM (Processed)	6680 images (1785 women)	Fujifilm, Hologic, Siemens, GE, IMS (11 sites)	ECNN	BI-RADS density (2 interpreting radiologists)	Yes	DSC = 0.77
Chang et al. [30]	FFDM (Raw)	108,230 images (21,759 women)	GE, Kodak, Fischer (33 sites)	ResNet-50	BI-RADS density (92 interpreting radiologists)	No	Four-class *K* = 0.67
Ciritsis et al. [31]	FFDM	20,578 images (5221 women)	N/R (1 site)	CNN	BI-RADS density (consensus of 2 interpreting radiologists)	No	AUC = 0.98 for MLO views AUC = 0.97 for CC views
Lehman et al. [32]	FFDM (Processed)	58,894 images (39,272 women)	Hologic (1 site)	ResNet-18*	BI-RADS density (12 interpreting radiologists)	No	Four-class *K* = 0.67
Mohamed et al. [33]	FFDM (Processed)	22,000 images (1427 women)	Hologic (1 site)	CNN AlexNet	BI-RADS density	No	AUC = 0.94
Mohamed et al. [34]	FFDM (Processed)	15,415 images (963 women)	Hologic (1 site)	CNN AlexNet	BI-RADS density	No	AUC = 0.95 for MLO views AUC = 0.88 for CC views
Haji Maghsoudi et al. [38]	FFDM (Raw)	15,661 images (4437 women)	Hologic (2 Sites)	U-net*	APD%	Yes	DSC = 92.5% in breast segmentation APD_diff_ = 4.2–4.9%
Li et al. [37]	FFDM (Raw)	661 images (444 women)	GE (1 site)	CNN	APD%	Yes	DSC = 76% in dense tissue segmentation
Kallenberg et al. [36]	FFDM (Raw)	N/R (493 women)	Hologic (1 site)	CSAE	APD%	Yes	DSC = 63% in dense tissue segmentation

The table describes the development image dataset used in each study, including format of mammographic images, sample size, and vendors, as well as methodological details for the AI model (output breast density measure, model architecture and availability of spatial density maps) and the model performance in breast density evaluation

*FFDM*: full-field digital mammography, *SM* 2D synthetic mammographic image acquired with digital breast tomosynthesis, *APD*% area percent density, *MLO* medio-lateral oblique, *CC* cranio-caudal, *cGAN* conditional generative adversarial network, *CNN* convolutional neural network, *ECNN *entirely convolutional neural network, *CSAE* convolutional sparse auto encoder, *DSC* dice score, *APD*_*diff*_ difference in APD%, *K* Cohen kappa coefficient, *AUC* area under the ROC curve, *Acc* accuracy

*Indicates publicly available AI model. *N/R* not explicitly reported in the paper

**Fig. 2:**
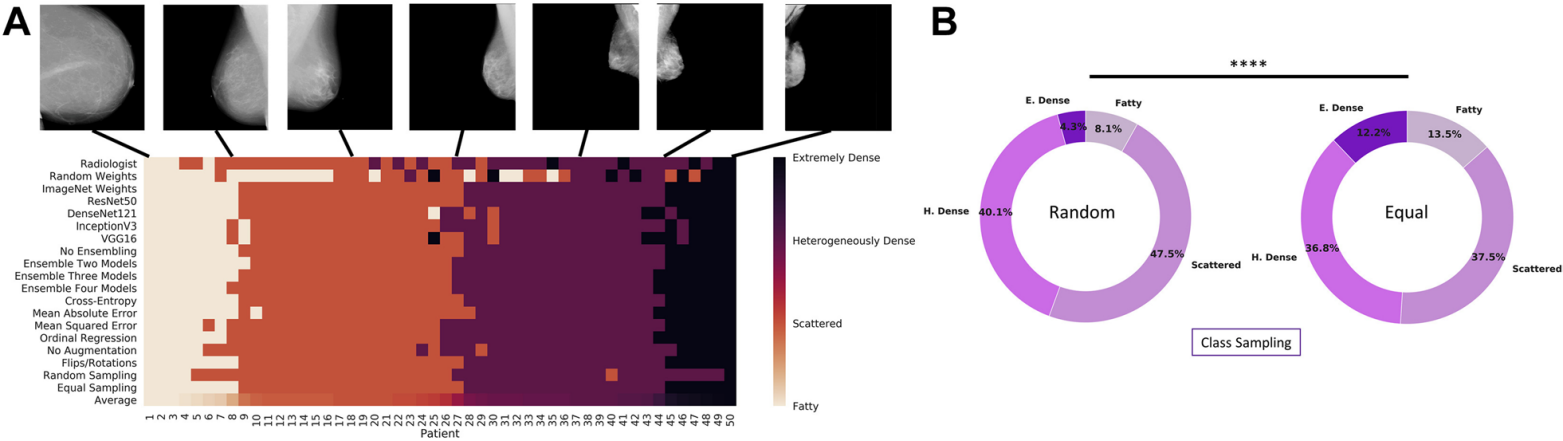
AI-based BI-RADS density classification. **A** A visual display of the range of BI-RADS density classifications for AI models trained with different architectures and training parameters for 50 patients in the testing set. The radiologist interpretation is displayed in the first row. The average breast density rating across all models and radiologist interpretations is displayed in the last row and was used to order the patients from least dense (left) to most dense (right). **B** The distribution of predicted breast density labels in the testing set differed for experiments with random class sampling (left) compared with equal class sampling (right) at each minibatch. ****P < .001. E. dense = extremely dense; H. dense = heterogeneously dense [30]. [Reprinted with permission from Elsevier (License Number: 5138920035119)]

Another important effort towards automating BI-RADS density classification via DL was based on a large multi-institution screening cohort of FFDM images from 21,759 women provided by the Digital Mammographic Imaging Screening Trial, which acquired the images from various FFDM vendors and the interpretations from 92 radiologists [30]. In addition to achieving good agreement with radiologists’ interpretations (four-class *K* = 0.67), this study explored the effects of different FFDM image formats (12-bit monochrome 1, 12-Bit Monochrome 2 and 14-Bit Monochrome 1), model architectures (ResNet-50, DenseNet-121, Inception-V3, and VGG-16) and training approaches (transfer learning, ensemble training, training set size, and cost functions) on the DL model performance (Fig. 2a). Furthermore, the study illustrated the difference between random sampling and equal sampling across each of the four BI-RADS categories as well as the decrease in performance when the format of FFDM images in the training set differs from that of FFDM images in the evaluation set (Fig. 2b).

Most recently, in what could be an essential step towards AI-enabled BI-RADS breast density assessment, research has focused on leveraging domain adaptation approaches to create DL models that utilize 2D synthetic mammographic (SM) images reconstructed from DBT acquisitions. The feasibility of this approach was demonstrated in large, racially diverse datasets from two clinical sites, where the adapted model achieved good agreement with the BI-RADS density classification from SM images by radiologists (four-class *K* = 0.72–0.79) [26]. Additional novel directions in this field include exploring state-of-the-art DL architectures [27, 28], as well as using federated learning, where participating institutions share model weights amongst themselves instead of the actual images. The aim of the latter approach is to train and improve DL models with large multi-institution cohorts [35].

**Fig. 3:**
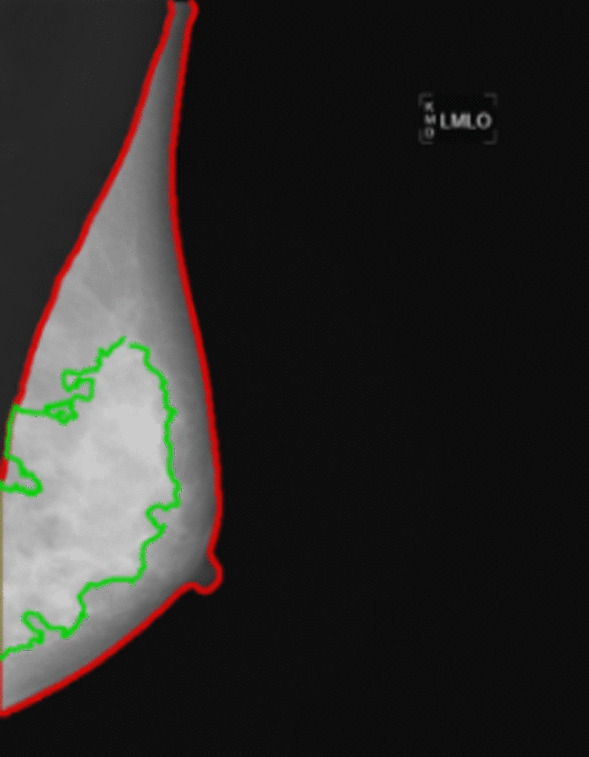
Example of AI-enabled density segmentation map from FFDM (estimated breast percent density, PD = 47%)

Despite the substantial progress made in automating BI-RADS density classification, merely striving for agreement with radiologists’ BI-RADS density interpretations is rather limiting since the rigid BI-RADS density categories do not capture finer density variations that may be important when refining breast cancer risk [21]. Moreover, radiologists’ BI-RADS density assessments reflect both the risk of developing breast cancer and the risk of masking in a single density evaluation when these components are two different tasks. Therefore, a key task for AI is to provide quantitative continuous measurements of breast density, to predict breast cancer risk, and to estimate the potential for masking of cancers due to areas of increased density [36–39] (Table 1). In one of the earliest AI studies in mammographic screening, Kallenberg et al. [36] introduced a DL method that first learned a feature hierarchy from unlabeled data and then used a classifier to estimate area percent density (APD) from raw FFDM images. The results of this study showcased high agreement between DL-based and manual dense tissue segmentations (Dice score, DSC = 63%), while in a case–control evaluation setting, the DL-based PD scores yielded an AUC of 0.59, which is competitive with reported AUCs from the literature on similar populations. In another study, Li et al. [37] proposed a supervised CNN approach to calculate APD from raw FFDM images. The proposed model achieved a Dice score of DSC = 76% for dense tissue segmentation and outperformed a traditional radiomic AI approach (DSC = 62%). Recently, “Deep-LIBRA,” built from a racially diverse set of cohorts from two clinical sites, was proposed as an AI-enabled method for estimating APD from raw FFDM images [38]. Deep-LIBRA demonstrated a mean Dice score of DSC = 92.5% for breast segmentation and a mean APD difference of 4.6% with respect to “gold-standard” human-rated Cumulus APD values. Moreover, in an independent blinded case–control evaluation [38], Deep-LIBRA yielded a higher case–control discrimination performance (area under the ROC curve, AUC = 0.61) than four other widely-used research and commercial breast density assessment methods (AUCs = 0.53–0.60). Besides providing continuous quantitative breast density measurements, the aforementioned AI methods also have the unique advantage of generating spatial density maps (Fig. 3). Such maps offer valuable insights about breast regions associated with limited mammographic sensitivity due to tumor masking.

As of now, breast density evaluation from 3D reconstructed image volumes has only been explored via traditional radiomic AI techniques [40] and no DL models have been extended to 3D DBT images. Moreover, with a few exceptions [26, 30, 38], most DL models have been developed using racially homogeneous datasets of FFDM images from a single vendor acquired at a single site, which may limit their ability to generalize to diverse breast cancer screening populations.

### AI developments in direct breast cancer risk assessment with mammographic images

Among the first to explore the potential of DL in breast cancer risk assessment, Kallenberg et al. [36] implemented a convolutional sparse autoencoder, which learned a hierarchy of increasingly abstract features from unlabeled data, as well as a simple classifier, which associated the learned features with breast cancer. Trained and tested on contralateral mammographic images of patients with unilateral breast cancer and matched healthy controls from two different databases, their method demonstrated promising case–control classification performance (AUC = 0.61 and AUC = 0.57, respectively). Another methodology was presented by Li et al. [41], where a pre-trained AlexNet model and feature extractor were applied to a set of FFDM images from two high-risk groups, i.e., BRCA1/2 gene-mutation carriers and unilateral cancer patients, as well as from healthy controls. Using a simple classifier, the authors showed that without any further fine tuning on mammographic images, the features from the first fully connected layer of the model could effectively discriminate healthy controls from both high-risk groups (AUC = 0.83 and AUC = 0.82 for BRCA1/2 gene-mutation carriers and unilateral breast cancer patients, respectively). Moreover, Gastounioti et al. [42] proposed a hybrid computational approach that employs CNNs to optimally fuse parenchymal complexity measurements generated by radiomic analysis into discriminative meta-features relevant for breast cancer risk prediction. Using a matched case–control dataset, Gastounioti et al. showed that CNNs can capture sparse, subtle, and relevant interactions between localized breast parenchymal patterns present in radiomic feature maps derived from mammographic images, thereby improving the breast cancer risk prediction of conventional parenchymal pattern analysis (AUC = 0.90 vs AUC = 0.79, *P* < 0.05).

**Fig. 4:**
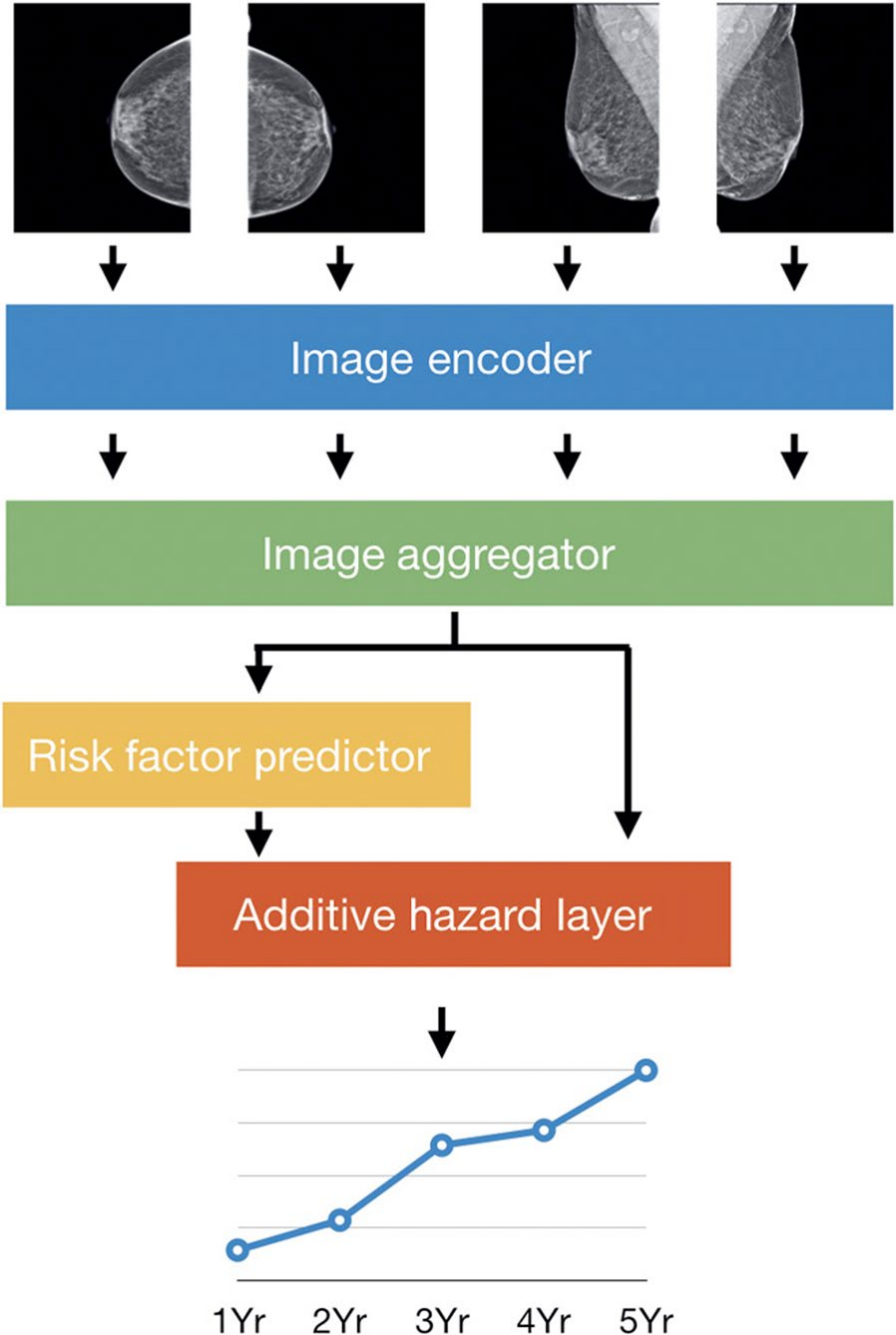
Use of the four standard mammographic views in long-term risk assessment via artificial intelligence [46]. [Reprinted with permission from The American Association for the Advancement of Science (License Number: 5138920821187)]

Additional studies [43–47] have focused on training DL models using large cross-sectional screening cohorts that represent the general screening population, with normal mammographic images acquired at least one year prior to the diagnosis of breast cancer or to negative (i.e., BIRADS 1 or 2) follow-up (Table 2). These study designs better conceptually reflect the task of breast cancer risk assessment, in the sense that clinically, one aims to identify high-risk women *before* an actual cancer is diagnosed (Fig. 4). Moreover, in such a study design, it is important to use breast cancer cases and controls of the same age or report age-adjusted evaluation measures, otherwise inflated performance estimates of risk prediction may result. The presented models have demonstrated promising performances with AUCs ranging from 0.60 to 0.84, often outperforming state-of-the-art breast cancer risk models [43, 44]. For instance, Ha et al. [47] found that an FFDM-driven DL risk score had greater predictive potential than BI-RADS breast density (odds ratios of 4.4 versus 1.7, respectively). Dembrower et al. [43] reported that their FFDM-driven DL risk score outperformed automated breast density measurements (odds ratios of 1.6 and 1.3, respectively). Last, Yala et al. [44] showed that a mammographic DL risk score outperformed the Tyrer-Cuzick model, which is used in clinical practice (AUC of 0.68 versus 0.62, respectively). Collectively, these studies provide preliminary evidence that FFDM-based DL models offer promise as more accurate predictors of breast cancer risk than density-based models and existing epidemiology-based models.

**Table 2:** Representative studies in AI-enabled direct breast risk assessment from mammographic images

Study	Image format	Time from exam to breast cancer diagnosis	# images (# women)	Vendors (# sites)	Model architecture	Model performance
*Long-term risk assessment*						
Yala et al. [46]	FFDM (processed)	1–5 years	295,002 images (91,520 women)	Hologic (3 sites)	ResNet-18*	AUC = 0.84, 1-year risk AUC = 0.76, 5-year risk
Dembrower et al. [43]	FFDM (processed)	3.6 ± 2.2 years	150,502 images (1188 cases; 10,563 controls)	Hologic (N/R)	Inception-ResNet*	OR = 1.55 OR_adj_ = 1.56 AUC = 0.65
Arefan et al. [45]	FFDM (processed)	1–4 years	452 images (113 cases; 113 controls)	Hologic (1 site)	GoogleLeNet	AUC = 0.68, CC AUC = 0.60, MLO
Yala et al. [44]	FFDM (processed)	1–5 years	88,994 images (1821 cases; 38,284 controls)	Hologic (1 site)	ResNet-18*	AUC = 0.68 for image only DL AUC = 0.70 for hybrid DL + risk factors
Ha et al. [47]	FFDM (processed)	2–5.3 years	N/R (210 cases; 527 controls)	GE (1 site)	CNN	OR = 4.42 Acc = 72%
*Short-term risk assessment*						
Lotter et al. [48]	FFDM (processed) DBT (MSP)	1–2 years	N/R (> 1000 cases; 62 K controls)	GE, Hologic (7 databases/sites)	RetinaNet*	AUC = 0.75–0.76
Eriksson et al. [49]	FFDM (processed)	3 months–2 years	N/R (974 cases, 9376 controls)	GE, Philips, Sectra, Hologic, Siemens (4 sites)	CNN**	HR = 7.9 AUC = 0.73
McKinney et al. [50]	FFDM (processed)	0 months–3.25 years	N/R (> 105 k women)	Hologic, GE, Siemens (4 sites)	RetinaNet MobileNetV2 ResNet-v2-50 ResNet-v1-50	AUC = 0.76–0.89

The table describes the development image dataset used in each study, including format of mammographic images, time window from mammographic exam to breast cancer diagnosis, sample size, and vendors, as well as model architecture and performance in breast cancer risk assessment

*FFDM* full-field digital mammography, *CNN* convolutional neural network, *AUC* area under the ROC curve, *Acc* accuracy, *OR* odds ratio, *HR* hazard ratio

*Indicates publicly available AI model. **Indicates commercial model. *N/R* not explicitly reported in the paper

In parallel with studies on long-term risk assessment, research groups have also explored the potential of AI in identifying women who are likely to be diagnosed with a cancer that was missed, masked, or fast growing [48–51] (Table 2). Eriksson et al. [49] developed a risk model that incorporates age, automated breast density, mammographic features (i.e., suspicious microcalcifications and masses) and bilateral parenchymal pattern differences detected by a DL-based commercial software. Lotter et al. [48] followed an annotation-efficient DL approach to develop a breast cancer detection model that can be applied to FFDM images as well as to 2D maximum suspicion projection (MSP) images generated from DBT reconstructed slices. McKinney et al. [50] designed an ensemble of three DL models, each operating on a different level of analysis (individual lesions, individual breasts and patient level) to produce a cancer risk score. Trained on large sets of mammographic images acquired around the time of breast cancer diagnosis or between subsequent screening exams, all three AI systems demonstrated promising predictive performance in short-term breast cancer risk assessment (AUC = 0.73–0.79).

Further research on decoupling inherent risk from early cancer signs and cancer masking [52], and assessing risk at various time points [46] is warranted, while also considering differences in screening intervals across different countries. Moreover, considering that a woman’s breast tissue changes over time and with various interventions (i.e., menopause, hormone replacement therapy, risk reduction surgery), developing methods that incorporate such sequential imaging data may further refine assessment of a woman’s individual risk over her lifetime of screening. Moreover, thus far, no DL models have been expanded for volumetric risk evaluation with DBT, which may yield further performance improvements.

### Technical challenges unique to mammographic imaging

Despite its vast potential in breast cancer risk assessment, AI is not a magic bullet and mammographic images present multiple technical challenges that go beyond fine-tuning the weights of a model. Most efforts to-date have focused on applying existing DL models to mammographic images rather than proposing new architectures specifically suited to this domain. However, developing a DL model for FFDM and DBT images requires more effort than just picking an “off-the-shelf” model that has been developed for natural images and training it on a large dataset. First, mammographic images are of much higher dimensionality than are typical natural images. To attempt to overcome this limitation, many research teams have heavily downscaled the original high-resolution mammographic image, e.g., from 2600 × 2000 pixels to 224 × 224 or 512 × 512 pixels. This is a common, effective approach in DL models for natural images where the object of interest usually occupies a large fraction of the image and what matters most is its macro-structure, comprising features such as shape and color. However, downscaling a high-resolution mammographic image may considerably impact the performance of a DL model, particularly in breast cancer risk assessment where subtle parenchymal patterns or microcalcifications associated with breast cancer risk may be lost [53]. Furthermore, mammographic imaging consists of two views for each breast: the CC view and the MLO view. In practice, radiologists usually consider a pattern more plausible if it is visible in both views. However, little attention has been devoted to this view-to-view correlation in DL approaches to breast cancer risk assessment [50, 53].

Additionally, DL models must be robust to the variation in mammographic images obtained by different technicians, vendors, and units. Normalizing mammographic images from different vendors is challenging because each vendor has its own proprietary post-processing software to make the FFDM images ready for presentation as well as in methods of reconstructing the individual DBT slices, especially since raw image data is not routinely stored. Considering that vendor-specific software is updated frequently, and image acquisition settings can change, the robustness of a DL model faces significant challenges. Consequently, harmonization and quality assurance of mammographic images are critical tasks that could potentially also be solved with AI techniques [54].

Despite the similarities between FFDM and DBT in terms of image acquisition [2], DBT poses more technical challenges compared to FFDM, particularly when it comes to simultaneously processing the numerous reconstructed DBT slices via 3D DL models. Reconstructed DBT volumes face two additional challenges, namely anisotropic voxels and a non-fixed number of slices that depend on compressed breast thickness (e.g., 45–90 slices with 0.09 × 0.09 × 1 mm resolution for Hologic DBT exams) and even overlap. Directly applying 3D convolutions to such images is challenging since it is hard for isotropic kernels to learn useful features from the anisotropic voxels and the capability of 3D networks is bounded by the GPU memory. Furthermore, due to a lack of large 3D image datasets, 3D DL models usually need to be trained from scratch, which can lead to unstable convergence and poor generalization issues. Therefore, extensive work is needed to develop DL architectures which are suitable for DBT, as well as to determine whether the knowledge, training data, and models developed for FFDM can be applied to DBT [26].

### Will AI tip the balance in breast cancer risk assessment?

This research field continues to rapidly evolve, and more mammography-based AI studies are being performed in breast cancer risk assessment. Such studies encompass different image data formats, DL model architectures, dataset sizes, and screening population characteristics; most interestingly, they have reported varying degrees of performance (Tables 1, 2). This large variability may raise concerns about the clinical applicability of AI-generated breast cancer risk scores and challenge our trust in them, especially when DL models lack the ability to explain the cause of their decisions [55]. However, when proper methodology has been employed, AI has demonstrated promising results and great potential to generalize across different datasets, rivaling and often improving on the performance of radiologists. Moving forward, we identify (a) reproducibility, (b) interpretability and (c) generalizability as three key priorities for AI in breast cancer risk assessment, with the goal of accelerating the translation of individualized AI-assisted risk stratification into routine breast cancer screening strategies.

Benchmarking efforts allowing the evaluation of the relative performance of different AI implementations for breast cancer risk assessment on the same datasets are essential to develop more robust and reproducible mammographic phenotypes of breast cancer risk. Currently, there are various publicly available FFDM databases for breast cancer detection (e.g., MIAS, DDSM and INbreast). Moreover, the “Digital Mammography DREAM Challenge” [56] and the “DBTex Challenge” [57] are important initiatives focusing on AI developments for breast cancer detection with FFDM and DBT images, respectively, with participation from several research teams around the world. These resources can also be useful in breast cancer risk assessment since using contralateral mammograms of patients with breast cancer is a common first-step in developing various mammographic phenotypes of breast cancer risk. This approach is based on the premise that a woman’s breasts—both affected and contralateral—share inherent breast tissue properties that predispose the woman to a certain risk of developing breast cancer [7]. However, public databases and benchmarking efforts with diverse FFDM and DBT data sets including imaging from years prior to a cancer diagnosis are needed [58, 59]. Moreover, numerous platforms are currently available to support comparative studies in AI research, including sharing code for training and evaluating a DL model (e.g., Bitbucket, GitHub and GitLab) as well as sharing DL models themselves, i.e. DL implementation along with learned weights (e.g., TensorFlow Hub and ModelHub.ai). In addition to improving reproducibility, such initiatives can significantly enhance the transparency and therefore, the trust, in AI algorithms, accelerating their transition into clinical implementation.

Interpretability is also key to advance AI applications in breast cancer risk assessment. DL models can only be debugged, audited, and verified when they can be interpreted. As such, interpretability is key to understanding the cause of an erroneous error or ensuring that causal relationships are picked up in a correct decision. A DL model that can sufficiently explain its decisions will not only gain users’ trust but will also identify data that is mislabeled or contains inconsistencies across institutions. This transparency and interpretability will facilitate improvements in quality control over training data. Interpretability methods may even serve as valuable discovery tools that identify new patterns and interactions in data. While so far, AI interpretability has focused mostly on image regions that drive the model’s decisions (commonly referred to as saliency maps), the set of available interpretability approaches is rapidly growing, offering unique opportunities for AI applications in mammographic images [60]. Even so, given the technical challenges of FFDM and DBT, adaptation of these methods to mammographic images will be methodologically challenging and will likely evolve into a whole new research field.

Another challenging step in establishing the role of AI in breast cancer risk assessment is validating that DL models generalize well to heterogeneous datasets [61, 62]. Therefore, large retrospective studies that include racially diverse breast cancer screening populations, different mammographic imaging machines, and various image acquisition settings are essential. Furthermore, while evaluation on retrospective datasets provides a “snapshot” of possible performance, the nuances of medical pathways cannot be underestimated. Therefore, in addition to large retrospective studies, prospective validation studies in real-time are essential to fully appreciate the performance of stand-alone AI applications, the influence of AI on radiologists’ performance, and the complex interaction between the two.

Finally, practical considerations related to clinical adoption of AI (e.g., IT infrastructure, upskilling of healthcare workforce, technical integration into clinical workflow, and radiologists’ engagement with AI), cost-effectiveness, and various ethical and legal dilemmas must be addressed before AI becomes common place in breast cancer risk assessment [13, 63]. In particular, the potential of AI to increase racial disparities in breast cancer screening must be carefully considered. Because it relies on retrospective screening data that often underrepresents certain minority groups and may contain biases, AI can could potentially exaggerate existing disparities for racial groups that already bear a high disease burden. Simultaneously, AI models may be less accessible to underrepresented groups, due to high cost, lack of insurance coverage, or limited availability (for example, in community sites versus academic tertiary care facilities). However, by carefully selecting underlying data and strategically deploying AI models within appropriate regulatory frameworks, AI risk models have the potential to help mitigate some racial disparities by offering equally accurate personalized breast cancer screening recommendations for all women and by reducing the number of cancers that are diagnosed at a later stage in some underrepresented groups.

## Conclusion

The rise and dissemination of AI in breast cancer screening is poised to improve breast cancer risk assessment and enable personalized screening recommendations. However, many technical challenges related to inherent properties of mammographic imaging are yet to be addressed, especially as AI developments transition to digital breast tomosynthesis. Furthermore, to accelerate the validation of AI breast cancer risk models and their transition into clinical implementation, it is paramount to enhance their reproducibility, interpretability, and robustness using large, heterogeneous datasets. With creative AI solutions to improve accuracy, validate performance, and cultivate trust in decision-making, AI will transform how breast cancer screening is performed.

## Abbreviations

AI: Artificial intelligence
ANNs: Artificial neural networks
BI-RADS: Breast imaging-reporting and data system
CC: Cranio-caudal
CNNs: Convolutional neural networks
DBT: Digital breast tomosynthesis
DL: Deep learning
MLO: Mediolateral oblique
ML: Machine learning
SM: 2D synthetic mammographic image
